# Leprosy of Ancient and Modern Times

**Published:** 1859-09

**Authors:** Robert Sim

**Affiliations:** Surgeon to the Anglican Hospital, Jerusalem, Syria


ART. V.—*Notes on the Nature of the Leprosy of Ancient and Modern Times.* By ROBERT SIM, M.D., Surgeon to the Anglican Hospital, Jerusalem, Syria.

THE question has often been asked whether the disease affecting those persons commonly called "Lepers," residing near the Zion Gate, is of the same nature as that described as "Leprosy" in the Mosaic code. Now, for reasons hereafter to be given, I do not think the "Tsarath" of Leviticus means any particular disease, but that it is the generic term for the severer forms of cutaneous affections in general. My friend Mr. Calman, whose knowledge of Hebrew entitles his opinion to confidence, takes this view of the case; and a very intelligent Rabbi, whom I have consulted, states that there are no fewer than *sixty-eight* kinds of leprosy. This is an enlargement on the "laws and tokens to discern leprosy," as laid down by Moses in the 13th chapter of Leviticus, but really more in accordance with the sense of the chapter and observation, than to assign the "Tsarath" to a single disease, as is done by some of the most eminent dermatologists of the present day.

The disease afflicting those poor creatures near Zion Gate is beyond all doubt—and as it is generally described the "Elephantiasis Græcorum" or "Lepra Araborum"—a tubercular disease, having many other names. With these, at present, I have nothing to do, as all I wish to prove is, that neither that nor any other *particular* disease is entitled to the definite appellation of "*the* leprosy." I need only recall, for a moment, that walls of houses and garments were liable to leprosies. It would be difficult to understand how either could be affected by tubercular diseases.

Mr. Calman says that the nitrous efflorescence, observable on the walls of the houses in this city, is a leprosy; also that cloth, made of diseased or badly-prepared wool, is still considered leprous by the Jews. Very probably moth-eaten clothes come under the same denomination.

A strong reason for maintaining that the "Elephantiasis Græcorum" is not *the* leprosy, is, that *all* the diseases described in the 13th chapter of Leviticus were *curable*, and that without the aid of miracle; for the *cleansing* of the leper by the *priest* was *subsequent* to the *healing* of the disease in the *patient*. By what means the lepers were cured is not of any consequence to know, even were it possible to determine; it is sufficient to know that they were cured without miracle. The "Elephantiasis Græcorum" is still, notwithstanding the progress of medical science, properly speaking an *incurable* disease, although when treated with energy at its beginning, it is sometimes checked. "Tsarath" is rendered by

Michaelis "Sharp affliction," and by Eben Esra, "Grave or Serious disease." Now, I believe that these terms might be applied to very many skin diseases besides the one in question. If *pain* be meant by these commentators, it would almost prove that the "Elephantiasis Græcorum" could not come under the denomination of "Tsarath," for one of its synonyms is "Elephantiasis Tuberculata ANAISTHETOS." Without laying too much stress, however, on this point, and knowing that the disease is occasionally accompanied by pain, it certainly deserves the title of "Sharp affliction," or "Grave disease." It will be observed that I do not say that it is not *a* leprosy, but simply that it ought not to be styled *the* leprosy.

The Rev. Dr. R. W. Stewart, of Leghorn, mentioned to me that he had seen a case, at Mount Sinai, of a man having the leg and thigh *quite white*. The man, if I did not misunderstand the doctor, exhibited himself *as a leper*. I have no doubt about his case being similar to that of Moses, Miriam, Gehazi, and Naaman. These cases were, in all probability, either "Lepra Vulgaris," or "Psoriasis Inveterata," both squamous diseases, and, to a person unaccustomed to see them, might readily be mistaken for the same. There is no doubt, as I believe Willan pointed out, that the term "leprosy" ought to be restricted to *squamous* diseases; but this proves nothing, either one way or another, as to the meaning of "Tsarath."

I believe all the confusion pervading the subject is referable to the loose manner in which the earlier translators performed their task.

I shall take each case mentioned in the Bible, and attempt, however unfit for the undertaking, to assign a name or class to it, in accordance with modern dermatology. Of course I do not pretend to say that it is possible to give each case a definite name, nor even to place it in a class; but I think it quite as reasonable to attempt this as to exclude *all* diseases in favor of the "Elephantiasis Græcorum" from the name of leprosy.

The cases as they occur are,—

1. That of Moses, Exodus iv. 6. This, as already stated, was probably "Lepra Vulgaris," or "Psoriasis Inveterata," (squamous.)

2. The case mentioned in 2d and 3d verses of 13th chapter of Leviticus may belong to almost any class of skin diseases, or even to no class. It would be endless to name the various diseases to which the description may be applicable.

3. The case in 7th and 8th verses of same chapter seems to have been either pustular or vesicular. If belonging to the first, probably a variety of "Impetigo," or "Ecthyma." If to the second, very likely "Eczema Simplex."

4. The case in 9th, 10th, and 11th verses might be almost any kind of ulceration,—from being "*old*," very probably an indolent varicose ulceration, which heals and breaks out again *ad infinitum*. It also might be the "Lichen Agrius," (papular.)

5. The case in 12th and 13th verses. This is a peculiar case, and seems to be "Lepra Vulgaris," or "Psoriasis Inveterata." The peculiarity consists in the leper being pronounced *clean* when completely covered by the eruption. Had this meant the mutilating malady observable at the Zion Gate, how different its termination!

6. The case in the 14th and 15th verses seems to be exactly like the fourth quoted.

7. The case in 19th and 20th verses may be a variety of "Impetigo," (pustular,) or "Eczema," (vesicular.)

8. The case in 21st and 22d verses may be "Lichen Simplex," (papular,) or "Lepra Nigricans," (squamous )

9. The case in 24th and 25th verses very probably "Eczema Rubrum," (vesicular,) or "Eczema Impetiginodes," combining, like many other cutaneous affections, the characters of vesicular and pustular diseases.

10. The case in 26th and 27th verses similar to the eighth case quoted.

11. The case in 29th and 30th verses is probably "Porrigo Decalvans" in the head, and "Sycosis Menti" in the beard, (both pustular.)

12. The case in 35th and 36th verses is probably "Porrigo Favosa," or "Porrigo Scutulata," (both pustular.)

13. The case in 42d, 43d, and 44th verses may be "Eczema of the Scalp," (vesicular,) or "Porrigo Decalvans," (pustular.)

14. The case of Miriam, Numbers, xii. 10, similar to first quoted.

15. The cases of Naaman and Gehazi, 2 Kings, v. 27, also similar to first case quoted.

16. The case of Azariah, 2 Kings, xv. 5. There is no description given, so if it be affirmed that the case was one similar to the "Lepra Araborum" of the present day, it can neither be proved nor disproved. His dwelling "in a several house" proves nothing.

17. The case of Uzziah, 2 Chronicles, xix. 20, *may* have been a case similar to the present "Lepra Araborum," or it may have been an obstinate case of "Porrigo Favosa," or any other disease of equal obstinacy.

I take no notice of the cases mentioned in the New Testament, as they have nothing to do with the subject, and are not described. Even if they were, it would have no interest, as it seems to me that the "laws and tokens" laid down in the 13th chapter of Leviticus are the *only* rules for "discerning" a leprosy.

I am quite aware of the imperfect manner in which I have performed my task; but all I wish to prove is, that neither the "Elephantiasis Græcorum," (Lepra Araborum,) nor any other *particular* disease, is entitled to the appellation of *the* leprosy. I might adduce many arguments in support of this opinion, but I think those already advanced are sufficient.

